# Effect of hydroxychloroquine on prevention of COVID-19 virus infection among healthcare professionals: a structured summary of a study protocol for a randomised controlled trial

**DOI:** 10.1186/s13063-020-04439-3

**Published:** 2020-06-03

**Authors:** Reihaneh Pirjani, Tahereh Soori, Ahmad Reza Dehpour, Mahdi Sepidarkish, Ashraf Moini, Arshia Shizarpour, Razieh Mohammad Jafari

**Affiliations:** 1 Department of Obstetrics and Gynecology, Arash Women’s Hospital, Tehran University of Medical Sciences, Tehran, Iran; 2 Department of Infectious Diseases, Arash Hospital, Tehran University of Medical Sciences, Tehran, Iran; 3 Department of Pharmacology, School of Medicine, Tehran University of Medical Sciences, Tehran, Iran; 4 Experimental Medicine Research Center, TUMS, Tehan, Iran; 5 Department of Biostatistics and Epidemiology, School of Public Health, Babol University of Medical Sciences, Babol, Iran; 6 Reproductive Epidemiology Research Center, Royan Institute for Reproductive Biomedicine, Tehran, Iran; 7 School of Medicine, Tehran University of Medical Sciences, Tehran, Iran; 8 Experimental Medicine Research Center, Tehran University of Medical Sciences, Tehran, Iran

**Keywords:** COVID-19, Randomised controlled trial, protocol, hydroxychloroquine, healthcare professionals, prevention

## Abstract

**Objectives:**

Comparison of the effect of hydroxychloroquine with placebo to prevent infection from the COVID -19 virus among healthcare professionals

**Trial design:**

Single centre, 2-arm, double-blind randomised (ratio 1:1) placebo-controlled trial

**Participants:**

Treatment staff who are in contact with patients and have at least 3 shifts a week in Arash hospital affiliated with Tehran University of Medical Sciences, in Iran and who consent to participate in the study. Exclusion criteria include: History of COVID -19 virus infection, clinical symptoms such as fever, nausea, dyspnea and myalgia in the past two months, history of underlying diseases hypersensitivity to hydroxychloroquine and G6PD enzyme deficiency.

**Intervention and comparator:**

Intervention group: Hydroxychloroquine 200 mg tablet of Amin Pharmaceutical. Control group: placebo which is completely similar in form and taste to 200 mg hydroxychloroquine tablet and is manufactured by the same factory (Amin Pharmacy). The dosage is two tablets daily, once a week for one to three months (based on the duration of the Coronavirus epidemic in Tehran).

**Main outcomes:**

Confirmed COVID-19 virus infection using Polymerase chain reaction (PCR) test is the primary outcome. The time period for measuring the primary outcome is any infection within the trial period up to one month after taking the last dose.

**Randomisation:**

The randomized block allocation method was developed using Stata version 15 software by an independent researcher, using a block size of six. Allocation to the two treatment groups will be conducted by this researcher using paper labels (random 10-digit codes) in a 1:1 ratio t The labels will be attached to the drug packages in order of randomization. Drug packages will be arranged in a box according to the randomization list.

**Blinding (masking):**

Participants and caregivers are blinded to group assignment and the data will be analyzed by an independent statistical expert who is unaware of the treatment allocation.

**Numbers to be randomised (sample size):**

A total of 282 participants will be randomised with 141 participants the Hydroxychloroquineeach intervention group and 141 participants to the placebo control group

**Trial Status:**

The protocol version number is 99-1-101-47091 and the approval ID is IR.TUMS.VCR.REC.1399.001 and recruitment began April 7, 2020, and is anticipated to be complete by August 7, 2020.

**Trial registration:**

The name of the trial register is Iranian registry of clinical trial (IRCT), registration number is IRCT20120826010664N6, date of trial registration is April 7, 2020,

**Full protocol:**

The full protocol is attached as an additional file, accessible from the Trials website (Additional file 1). In the interest in expediting dissemination of this material, the familiar formatting has been eliminated; this Letter serves as a summary of the key elements of the full protocol.

## Supplementary information

**Additional file 1.** Full study protocol.

## Data Availability

Not applicable.

