# Supplementary material for: Effect of hydroxychloroquine on prevention of COVID-19 virus infection among healthcare professionals: a structured summary of a study protocol for a randomised controlled trial
Source: Trials. 2020 Jun 3;21:467. doi: 10.1186/s13063-020-04439-3 (PMC7267750; doi:10.1186/s13063-020-04439-3)
Supplement: Supplementary file 1 — Additional file 1. Full study protocol. [file 13063_2020_4439_MOESM1_ESM.docx]

**Effect of hydroxychloroquine on prevention of covid-19 virus infection among healthcare professionals** **: A structured summary of a study protocol for a randomised controlled trial**

**Introduction**

The Covid-19 virus, caused by a new mutation in the coronavirus, has become pandemic all around the world in recent months. Due to the high risk of transmission of the virus along with the treatment of infected people, prevention of infection in healthy people is one of the most important priorities at this time. Studies have shown that one of the pathogenic mechanisms of the coronavirus is its binding to the ACE2 receptor in body tissues, especially the human lung. A study in China found that the ACE2 receptor is the main receptor for the Covid-19 virus in the human lung.Hydroxychloroquine is used in a variety of mechanisms to treat infectious diseases and autoimmune diseases. One of the mechanisms of this drug is to prevent the coronavirus from binding to the receptor. In a 2006 study of the association between anti-infective drugs and the SARS virus, hydroxychloroquine was cited as one of the treatments for the virus, and its mechanism was described as interference with the final glycosylation of the ACE2 receptor.

At present, there is no consensus about the use of hydroxychloroquine on the prevention of Covid 19 virus, and unfortunately it is sometime used as a prophylactic method without strong scientific evidence. Therefore, in this study, we decided to compare hydroxychloroquine with placebo in prevention of coronavirus infection among treatment staffs.

**Method**

In this Single centre, 2-arm, double-blind randomised (ratio 1:1) placebo-controlled trial, treatment staffs in Arash hospital affiliated with Tehran University of Medical Sciences will be invited to participate in the study. Ethics committee of Tehran University of Medical Sciences has approved the study, its ethical code is IR.TUMS.VCR.REC.1399.001 and approval was granted on march 22 , 2020

Healthcare professionals will be enrolled in the study based on inclusion and exclusion criteria. Inclusion criteria: Staffs who are in contact with patients and have at least 3 shift a week in the hospital and consent to participate in the study. Exclusion criteria: History of coronavirus infection or clinical symptoms such as fever, nausea, dyspnea and myalgia in the past two months, history of underlying diseases, hypersensitivity to hydroxychloroquine, G6PD enzyme deficiency. After obtaining informed consent, they will be randomized into two groups. Intervention group: Hydroxychloroquine 200 mg tablet of Amin Pharmaceutical. Control group: placebo which is completely similar in form and taste to 200 mg hydroxychloroquine tablet and is manufactured by the same factory (Amin Pharmacy). The dosage is two tablets daily, once a week for one to three months (based on the duration of the Coronavirus epidemic in Tehran). Confirmed COVID-19 virus infection using Polymerase chain reaction (PCR) test is the primary outcome. The time period for measuring the primary outcome is any infection within the trial period up to one month after taking the last dose.

**Randomisation**

The randomized block allocation method will be conducted by an independent researcher using a random number sequence, generated with a computer-generated randomization scheme, according to a randomized block design. The block size was six. . Allocation to the two treatment groups will be conducted by this researcher using paper labels (random 10-digit codes) in a 1:1 ratio. The labels will be attached to the drug packages in order of randomization. Drug packages will be arranged in a box according to the randomization list.

**Blinding (masking)**

Participants and caregivers are blinded to group assignment and the data will be analyzed by an independent statistical expert who is unaware of the treatment allocation .Both hydroxychloroquine and placebo tablet are identical in appearance, smell and taste.

**Numbers to be randomised (sample size)**

It was assumed that a total sample of 282 subjects (141subjects per group), which included a 15% dropout factorwould provide 80% power to detect a difference in proportion of the occurrence of COVID-19 between the hydroxychloroquine and placebo groups. Assuming 15% of the disease occurrence in placebo group and 5% in the hydroxychloroquine group and a two-sided test having a type I error of 0.05.

**Trial Status**

The protocol version number is 99-1-101-47091 and the approval ID is IR.TUMS.VCR.REC.1399.001 and recruitment began April 7, 2020, and is anticipated to be complete by August 7, 2020.

**Trial registration**

The name of the trial register is Iranian registry of clinical trial (IRCT), registration number is IRCT20120826010664N6, date of trial registration is April 7, 2020,
